# Microencapsulated drug delivery: a new approach to pro-inflammatory cytokine inhibition

**DOI:** 10.3109/02652048.2012.658443

**Published:** 2012-02-21

**Authors:** Carl W. Oettinger, Martin J. D'Souza

**Affiliations:** College of Pharmacy and Health Sciences, Mercer University, 3001 Mercer University Drive, Atlanta, GA 30341, USA

**Keywords:** sepsis, TNF, antisense, oligonucleotides, NF-kappaB, antioxidant, catalase, endothelium, phagocytosis

## Abstract

Context: This article reviews the use of albumin microcapsules 3–4 mm in size containing cytokine inhibiting drugs which include neutralizing antibodies to TNF and IL1, CNI-1493, antisense oligonucleotides to TNF and NF-kappaB, and the antioxidant catalase. Objective: Describe the effects, cellular uptake and distribution of microencapsulated drugs and the effect in both a peritonitis model of infection and a model of adjuvant-induced arthritis. Methods: The studies performed by our group are reviewed, the only such studies available. Results: Microencapsulation of these compounds produced high intracellular drug concentrations due to rapid uptake by phagocytic cells, including endothelial cells, without toxicity. All compounds produced excellent inhibition of TNF and IL1 resulting in improved animal survival in a peritonitis model of septic shock and inflammation in an arthritis model. Conclusion: Albumin microencapsulated pro-inflammatory cytokine inhibiting compounds are superior to equivalent concentration of these compounds administered in solution form.

## Introduction

Microencapsulated drug delivery is a novel approach to targeting phagocytic cells. Microencapsulated drugs have the potential to improve the physiologic and therapeutic activity of compounds by intracellular delivery. Reduced toxicity and prolongation of the effectiveness of a drug may also result. Medications exert their effects by either acting on a cell wall receptor or penetrating the cell wall to exert an effect on an intracellular metabolic or immunologic process. Targeting a phagocytic cell whose function is to engulf particulate matter is a method to deliver a drug dispersed in a non-toxic substance (species specific albumin) directly into the cell (Bender et al., 1994; Shafer et al., 1994). The higher concentration of intracellular drug may result in a greater physiologic effect. Phagocytic cells include macrophage/monocytes, polymorphonuclear cells, endothelial cells and dendritic cells. These cells have overlapping biological functions, which include cytokine synthesis, immunologic regulation, reactive oxygen species (ROS) generation and metabolism, cell barrier function and elimination of infectious organisms. In addition to these functions, endothelial cells play a vital role as a selective transport barrier in the circulatory system. Microencapsulated delivery of a compound has the potential to greatly increase the intracellular concentration of the drug and thus improve the biologic effectiveness. We have microencapsulated antibodies, small water soluble molecules, antisense oligonucleotides and large enzymes such as catalase, in producing accentuated physiologic effects in pro-inflammatory cytokine inhibition.

The pro-inflammatory cytokines play a major role in the inflammatory response of many conditions such as sepsis, rheumatoid arthritis and inflammatory bowel disease (Andreakos and Feldman, 2003). TNF and IL1 have wide ranging immediate and profound physiologic effects. TNF is synthesized within minutes of endotoxin exposure and reaches a peak concentration in several hours both *in vitro* and *in vivo* models (Michie et al., 1988; Oliver et al., 1993). IL1 synthesis reaches peak levels 24 h after endotoxin stimulation using *in vitro* methods (Oliver et al., 1993). Both cytokines have overlapping physiologic effects such as hypotension, increased vascular permeability, migration of white blood cells through vascular walls, fever and increased protein catabolism. When IL1 and TNF are given together to experimental animals, the hypotensive effect is synergistic (Okusawa et al., 1988). There is much experimental evidence implicating these cytokines in the pathogenesis of septic shock in experimental animals. However, no single experimental model of sepsis completely mimics the human septic state. TNF neutralizing antibodies when administered in numerous clinic trials of septic shock have not demonstrated improved patient survival (Dinarello, 2010). There have been numerous TNF antibody preparations which have proven beneficial in the treatment of rheumatoid arthritis. It is tempting to speculate that the optimal effective delivery vehicle to enhance the inhibition of cytokine synthesis in sepsis and other conditions have not yet been clinically evaluated.

It is the purpose of this review to summarize recent developments in the methodology and biologic effects of albumin microencapsulated drug delivery. A summary of the physiologic effect of the inhibition of TNF and IL1 by microencapsulated compounds is described in the experimental models of sepsis and rheumatoid arthritis.

## Production of albumin microspheres

**Figure 1:**
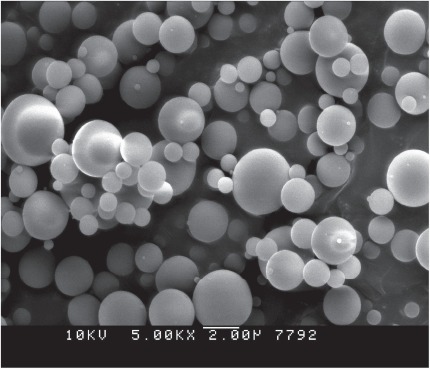
Scanning electron micrograph of albumin microspheres (magnification ×5500), size 1–7 μm.

The microspheres produced in our laboratory for drug delivery are particles ranging from 3 to 4μm in size composed of a matrix of species specific albumin (Figure 1; Gayakwad et al., 2009). In brief, water soluble drugs are combined with an aqueous solution of the drug and albumin and sprayed with a micronebulizer into olive oil producing a microemulsion. The albumin is chemically linked with glutaraldehyde and the excess glutaraldehyde is neutralized with sodium bisulphate before the active drug is added. The process produces hardened microcapsules 3–4μm in size containing the drug dispersed through-out the albumin matrix of the microcapsule. These microcapsules are smaller then the cellular components of blood and have produced no evidence of capillary occlusion. The distribution of the drug allows continued intracellular release of the drug as the microcapsule is degraded by intracellular proteolytic enzymes. The microcapsules are then desiccated and stored for use. The complete details of drug loading and microsphere characteristics for antisense oligonucleotide to NF-κB, dexamethasone and the antioxidant enzyme catalase are found in recent publications (Siwale et al., 2008; Gayakawad et al., 2009; Uddin et al., 2011).

### Microsphere physiologic characteristics

**Figure 2:**
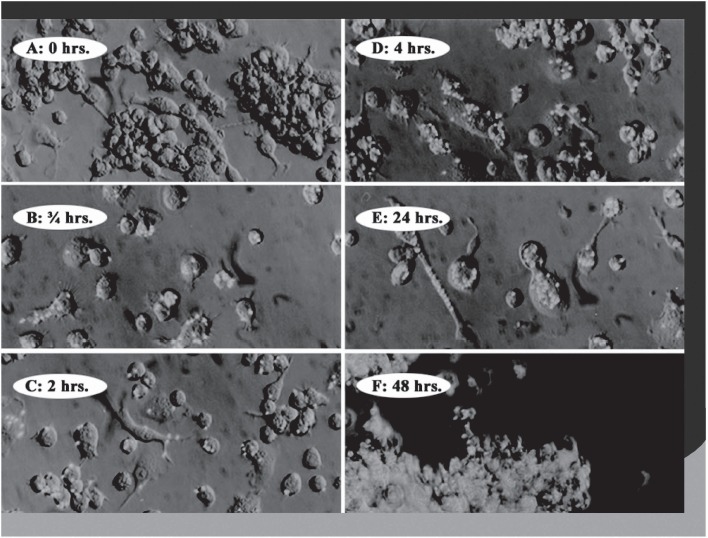
Serial photographs of fluorescein-labelled albumin microspheres incubated with macrophages. Progressive phagocytosis of the microspheres is seen.

Phagocytic cells are natural targets for microencapsulated drug delivery. Cells such as macrophages/monocytes, polymorphonuclear and endothelial cells rapidly phagocytoze albumin microcapsules both *in vitro* and *in vivo* (Figure 2). *In vitro* studies have shown that within 1 h, 70% of a dose of I-125 radiolabelled microencapsulated IL1 was taken up by peritoneal macrophages (Oettinger et al., 1999). Other studies utilizing whole blood demonstrated that in 2 h, individual macrophages had phagocytozed as many as five microcapsules (Oettinger and D'Souza, 2003). After injection of microcapsules into the blood stream of an experimental animal, less than 2% of the injected dose was detected in the circulation in 5 min (D'Souza and D'Souza, 1995).

Macrophages are present in the liver (kupfer cells), lung (pneumocytes), spleen, kidney, CNS and other organs. Macrophages play unique roles in each organ but have common functions of pro-inflammatory cytokine release to initiate the inflammatory process, phagocytosis of micro-organisms and cellular debris and communication with other immune cells. Prompt cytokine synthesis (TNF, IL1) by macrophages, endothelial cells and white blood cell is a major response in both local and systemic inflammatory conditions. In addition, these cells produce hydrogen peroxide and other ROS as part of the respiratory burst, an important mechanism in bacterial removal. ROS such as hydrogen peroxide activate NF-κB, the nuclear transcription factor initiating cytokine synthesis (de Oliveira-Marques et al., 2007). The inflammatory response resulting from cytokine synthesis leads to vascular changes in permeability and amplification of the inflammatory process.

**Figure 3:**
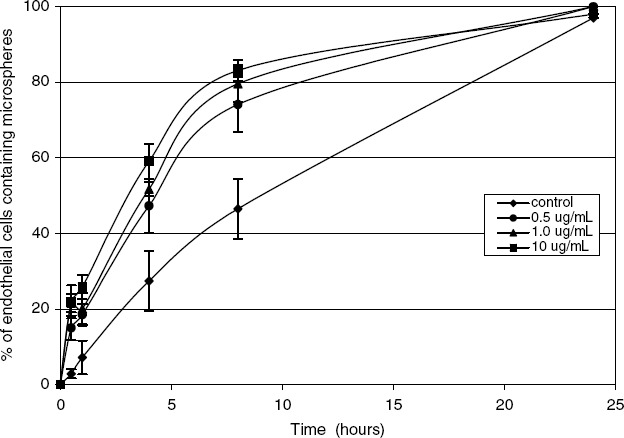
Endothelial cells were incubated with endotoxin. Fluorescein-labelled albumin microspheres were added and the percentage of cells containing microspheres was determined after 0.5, 1.0, 4.0 and 24 h. *p < 5 0.05 compared to the control group. Increased microsphere uptake by phagocytic cells was noted after endotoxin exposure.

Endothelial cells are phagocytic and rapidly engulf albumin microspheres *in vitro* (Zhaowei et al., 2007). A total of 28% of endothelial cells phagocytoze micrcapsules *in vitro* in 5.5 h. By 24 h, 97% of endothelial cells had phagocytozed microcapsules. After exposure to endotoxin, 47% of endothelial cells phagocytozed microcapsules by 5.5 h (Figure 3). The endothelium is a particularly important cell type as it forms the barrier between the circulation and the interstitial space. The endothelial cells comprising the blood vessels are responsible for the transfer of nutrients, oxygen, vascular resistance regulating blood pressure and the regulation of blood flow. These cells through alterations in permeability and resistance play a major role in regulating metabolism. Endothelial cells synthesize cytokines such as TNF, ROS and metabolize lipids. In our laboratory, we have demonstrated rapid endothelial uptake of microencapsulated antisense oligonucleotides to NF-κB (Zhaowei et al., 2007). After phagocytosis of NF-κB containing microcapsules, TNF synthesis by endothelial cell was inhibited by 78% after endotoxin stimulation. This resulted in reversal of increased endothelial-induced permeability using an *in vitro* model of endothelial cell permeability. Endothelial cells comprise one of the largest cell types in the body. Thus, the regulation of these functions can be crucial in controlling many inflammatory diseases affecting the endothelium such as the systemic inflammatory response seen in sepsis.

The effect of clodronate microspheres in resident ED1 positive macrophages. The tissue sections containing ED1 macrophages were stained using an immunoperoxidase technique. Panel A macrophages (stained brown) in the spleen of a healthy control rat (magnification × 100); panel B 72 h after treatment with clodronate microspheres. Splenic macrophages are 490% depleted. Panel C: Kupfer cells (stained brown) in the liver of a healthy control rat. Panel D: 72 h after treatment with clodronate microcapsules. Kupfer cells are >90% depleted.

**Figure 4:**
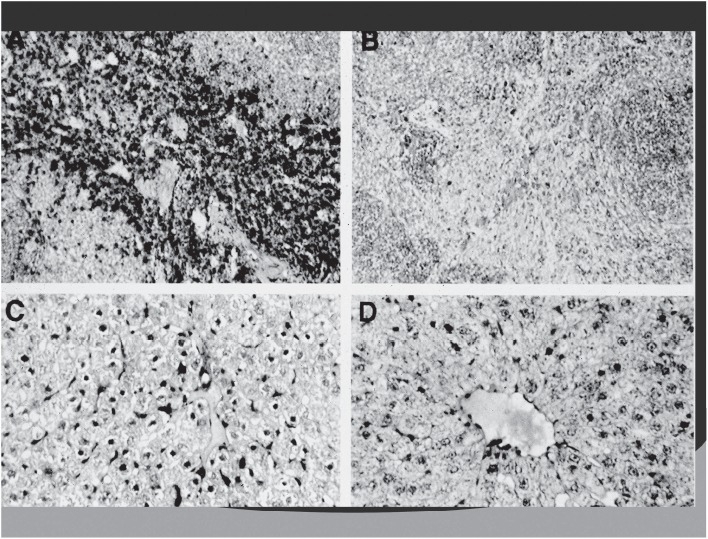
Scanning electron micrograph of albumin microspheres (magnification ×5500), size 1–7 μm.

Clodronate, which is a bisphosphonate, was encapsulated into albumin microspheres and administered intravenously to normal rats. Microencapsulated clodronate causes cellular death of macrophages after phagocytosis of the microcapsule (Van Rooijen et al., 1990). Macrophages in tissue and blood were documented by histologic staining for a macrophage antigen ED-1. Macrophages were decreased by greater than 90% in liver, spleen, blood and kidney (Figure 4; D'Souza et al., 1999). This observation demonstrates that there is high organ penetration and uptake by macrophages of the albumin microcapsules.

The microcapsules contained in macrophages are also attracted to areas of infection, presumably under the influence of macrophage chemoattractant factors (D'Souza et al., 1999). Microspheres containing radio-labelled IL1 antibody were given to rats with peritonitis. A significant increase in peritoneal activity of the microencapsulated radio-labelled neutralizing antibody to IL1 suggested localization of the microsphere containing antibody to the infected area (Oettinger et al., 1999).

Phagocytosis of a microencapsulated drug also increases the duration of action. CNI-1493, a potent cytokine inhibitor, was microencapsulated and given to a model of endotoxic shock in the rat (D'Souza et al., 1999). This resulted in a 100% survival of the infected rats. When microencapsulated dose of CNI-1493 was given follow by a fatal dose of *Escherichia coli* endotoxin 24 h later, 83% survival was noted. This indicates continued effectiveness of the drug in cytokine inhibition due to persisting intracellular concentrations of CNI-1493 sufficient to inhibit cytokines after a severe physiologic challenge. In addition, the duration of action of the microencapsulate antisense oligonucleotides to NF-κB was observed to be at least 3 days in duration when given to primates.

In summary, microencapsulated drug delivery to phagocytic cells has resulted in: (1) efficient inhibition of pro-inflammatory cytokine synthesis, (2) penetration of microencapsulated drug delivery to resident macrophages in solid organs, (3) high intracellular concentrations of cytokine inhibiting compounds, (4) migration of microen-capsulated drug to the area of infection and (5) endothelial cell uptake with cytokine inhibition and partial reversal of endotoxin induced increased permeability.

## Experimental models

The experimental models employed in the evaluation of pro-inflammatory cytokine synthesis have been endotoxic shock, sepsis (both Gram-positive and Gram-negative organisms), and the adjuvant induced (*Mycobacterium butyricum*) arthritis model of rheumatoid arthritis. Much experimental evidence has been accumulated implicating both TNF and IL1 in the pathogenesis of sepsis (systemic inflammatory response syndrome). TNF and IL1 are both 17 kDa cytokines synthesized immediately after the administration of either endotoxin (*E. coli* 15 mg/kg) or live organisms as described earlier. Many experimental models have been used to evaluate the effect of blocking these cytokines on the effect of sepsis. This topic has been recently reviewed (Doi et al., 2009; Zarjou et al., 2011). There is general agreement that no single experimental model has proven satisfactory to totally replicate human sepsis. However, compartmentalized infection models such as peritonitis are useful in evaluating the effectiveness of therapeutic delivery systems. In a model described by Bagby et al. (1991), *E. coli* (10^10^ colony forming units) is injected directly into the peritoneal cavity of rats. Much greater levels of TNF were produced in the peritoneal cavity compared to the systemic circulation. The administration of TNF antibodies is effective in reversing the mortality when endotoxin is injected directly into the systemic circulation but have no effect on mortality on those animals with peritonitis. Presumably, the antibodies do not adequately penetrate into the area of compartmentalized infection. This example would be similar to human infection which is frequently compartmentalized as well (pneumonia, intraperitoneal infection). To our knowledge, there have been no other reports of survival in this model utilizing cytokine inhibiting drugs other than reported by our group. Thus, intraperitoneal infection is an ideal approach to evaluate the effectiveness of new therapeutic compounds in treatment. We have administered antibiotics in this model as infection could not clear without specific antimicrobial treatment. However, antibiotics given alone had no appreciable effect on animal survival.

Rheumatoid arthritis is thought to be a TNF dependent form of arthritis affecting the synovial membrane of joints in both experimental animals and humans (Smith-Oliver et al., 1993; Szekanecz et al., 2001).

**Figure 5:**
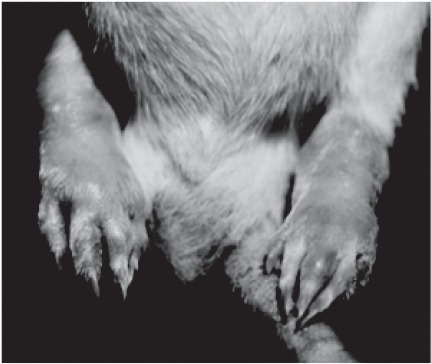
Development of arthritis and tissue swelling in rats after subcutaneous injection with *M. butyricam*.

The clinical use TNF neutralizing antibody preparations have revolutionized the treatment of this crippling disorder (Moreland et al., 1997). A classic animal model for this disease is rats being given subcutaneous *M. butyricum* (Ward and Cloud, 1966). The rats develop a picture of joint inflammation (Figure 5). We have used this model to evaluate the effect of microencapsulated antisense oligomers to NF-κB (Akhavein et al., 2008).

### Microencapsulated compounds inhibiting cytokine synthesis

Our research has concentrated on drugs which have an effect on inhibiting the synthesis of pro-inflammatory cytokines. Endotoxin and infectious organisms are some of the most potent stimulators of the cytokine cascade. The microencapsulated compounds that we have evaluated are neutralizing antibodies to TNF and IL1, CNI-1493, antisense oligonucleotides to TNF and NF-κB, the antioxidant enzyme catalase and dexamethasone. All of these micro-encapsulated compounds have proven effective in TNF and IL1 inhibition with improved animal survival in a peritonitis model of septic shock.

### Cytokine neutralizing antibodies

Neutralizing antibodies to TNF have been used to inactivate specific cytokines in both experimental conditions and more recently in clinical disease states such as rheumatoid arthritis (Choy and Panayi, 2001). These antibodies combine with the specific cytokine to neutralize the physiologic effect of the cytokine. The preparations are high molecular weight and may not easily penetrate into inflamed tissues. In previous animal models, these antibody preparations administered in solution form had no effect in survival in experimental models of compartmentalized infection such as peritonitis (Bagby et al., 1991). Microencapsulation of antibodies to TNF and IL1 greatly improved TNF and IL1 inhibition *in vitro* and survival in experimental models of peritonitis using either *S. aureus* or *E. coli* (Oettinger et al., 1999; D'Souza et al., 2000). The combination of both micro-encapsulated neutralizing antibodies to TNF and IL1 was most effective, producing 100% survival. These microen-capsulated antibody preparations were effective even when administered 4 h after the infectious organisms were given. This time point is significant as it is the time of peak TNF release in this model. This demonstrates that the microencapsulated neutralizing antibodies were effective even when TNF was at its highest concentration in response to the infectious organisms. The proposed mechanism for the improved effectiveness is a greater degree of cytokine inhibition by the intracellular delivery of antibody, high levels of tissue penetration to resident phagocytic cells, and microsphere attraction to inflamed areas as a result of chemoattractive factors (Leonard and Yoshimura, 1990).

### Water soluble compounds (CNI-1493)

Low molecular weight water soluble compounds are ideally suited to microencapsulation using an albumin matrix. CNI-1493 is a water soluble guanylhydrazone compound with a molecular weight of 890. CNI-1493 has been shown to be a potent inhibitor of TNF synthesis by inhibiting post-translational synthesis of TNF (Bianchi et al., 1995, 1996). Microencapsulation of CNI-1493 followed by *E. coli* endotoxin 15 mg/kg resulted in 100% survival. The peritonitis model using *E. coli* organisms had an 83% animal survival in 5 days in an otherwise fatal infection. Both TNF and IL1 were markedly inhibited. The duration of action of the microencapsulated CNI-1493 was 24h as previously described (D'Souza et al., 1999).

### Oligonucleotides

Antisense oligonucleotides are large molecules composed of nucleotide bases which can combine with specific sequences of mRNA in the nucleus to prevent the translation of a specific protein synthesis by the ribosome. Antisense oligonucleotides have been constructed which will inhibit the protein synthesis of both TNF and NF-κB (Oettinger and D'Souza, 2003; D'Souza and Oettinger, 2005). Both of these antisense compounds have been microencapsulated with excellent success in TNF and IL1 inhibition to both endotoxin stimulation and septic shock (Oettinger and D'Souza, 2003; D'Souza and Oettinger, 2005). The intracellular concentration of the antisense oligonucleotide to NF-κB was determined to be significantly greater in the microencapsulated formulation than solution in both macrophages and endothelial cells (Zhaowei et al., 2007). Antisense oligonucleotides to NF-κB have been used in the primate model and found to inhibit pro-inflammatory cytokine synthesis (TNF, IL1 and IL6) greater than 90% after *ex vivo* whole blood endotoxin stimulation for 3 days (Oettinger et al., 2007). These microen-capsulated oligomers were found to be non-toxic and non-antigenic in the primate. Antisense oligonucleotides to NF-κB have also been used to inhibit the inflammatory response in a rodent model of rheumatoid arthritis (Moreland et al., 1997). After the induction of arthritis in rats, microencapsulated antisense oligonucleotides to NF-κB were administered intraperitoneally for 2 weeks. An 82% reduction in rat paw volume was observed 15 days after injections of 7.5 mg/kg of microencapsulated oligomers to NF-κB. The long-term use of microencapsulated antisense to NF-κB was non-toxic and had no observable allergic reactions in the rat.

### Glucocorticoids

Dexamethasone has been shown to be a powerful inhibitor of NF-κB and the inflammatory process (Scheinman et al., 1995; De Bosscher et al., 1997). However, the systemic administration of glucocorticoids is associated with well-known systemic side effects. Microencapsulated dexamethasone has been shown to inhibit TNF by 84%, IL1 by 93%, IL6 by 83% and IL8 by 81% after stimulation with endotoxin in a whole blood endotoxin mode (Uddin et al., 2011). This inhibition was significantly more effective in cytokine inhibition than the equivalent dose of dexamethasone given in solution. Microencapsulated dexamethasone given in combination with antibiotics resulted in 90% survival in a peritonitis model compared to 30% in a group, given an equivalent amount of dexamethasone in solution with antibiotics. Thus, microencapsulated dexamethasone is an excellent inhibitor of pro-inflammatory cytokines.

The *in vitro* effectiveness of microencapsulated dexamethasone also produced marked improvement in animal survival using a severe model of infection. Microencapsulated dexamethasone may cause less systemic side effects in animals than dexamethasone in solution.

### Antioxidants

Much recent work has implicated ROS in many disease processes (Pantano et al., 2006; Alun et al., 2008). The production of hydroxyl ion, hydrogen peroxide and other by-products of aerobic metabolism when produced in excess, have been associated with tissue damage to nucleic acids, lipids and other cellular constituents. Septic shock, ischemia/reperfusion situations, atherosclerosis along with many other inflammatory clinical conditions are associated with ROS overproduction. Phagocytic cells produce ROS as a part of the respiratory burst (Iles and Forman, 2002). Recent work in our laboratory has reported the microen-capsulation of the antioxidant catalase (Siwale et al., 2009). Catalase is a naturally occurring enzyme present in the peroxisomes of nearly all cells. Catalase has an enormous capacity to degrade hydrogen peroxide into O_2_ and H_2_O. In cell culture models using endothelial cells, microencapsulated catalase inhibited hydrogen peroxide production, nitrate synthesis and TNF release. The intracellular catalase was seven-fold higher using microencapsulation. In an *in vivo* endotoxic shock model, TNF was significantly inhibited and there was a survival rate of 60% in an otherwise fatal experimental model (Siwale et al., 2009). The addition of antisense oligonucleotides to NF-κB improved the survival rate to 80% with synergistic inhibition of TNF. These results conclusively demonstrate that intracellular delivery of antioxidants using albumin microcapsules can produce a favourable biologic effect.

### Additive and synergistic effects in cytokine inhibition

The intracellular delivery of cytokine inhibiting drugs resulting in high intracellular concentrations make it possible that drugs inhibiting cytokine synthesis by different mechanisms may be beneficial. The combination of neutralizing antibodies to TNF and IL1 produced improved survival and pro-inflammatory cytokine inhibition in the model of peritonitis (11). TNF and IL1 are similar in molecular weight and function but have different time release profiles in sepsis. Using the whole blood model, TNF reaches peak concentrations after endotoxin stimulation in 2 h. IL1 synthesis begins after 1 h and continues to rise for 24 h. Thus, the combination of microencapsulated neutralizing antibodies would act at different stages of the inflammatory response.

The combination of CNI-1493 and antisense oligonucleotides to NF-κB produced synergistic effects in survival and additive effects in TNF inhibition (Oettinger and D'Souza, 2010). In this study, doses of both compounds were used that were ineffective alone in influencing survival in the peritonitis model. The combination produced 60% survival indicating synergism in the effects of the drugs. CNI-1493 acts by inhibition post-translational TNF synthesis, antisense oligonucleotide to NF-κB inhibits pro-inflammatory cytokine at the translational level. The combination of the microencapsulated antioxidant catalase and antisense oligonucleotides to NF-κB was additive in both animal survival and TNF inhibition in the septic model (Siwale et al., 2009).

### Future direction

These studies have demonstrated excellent results in the treatment of a severe compartmentalized model of sepsis. However, there is no single model of sepsis that mimics completely the human septic state which is widely varied in clinical presentation. In addition, humans have diverse co-morbid conditions not present in experimental animals that are usually young and free of known disease states. Thus, it may be only possible to evaluate this promising method of drug delivery in a clinical trial after appropriate pre-clinical evaluation. No toxicities or allergic reactions have been detected in both rats and primates. The excellent results observed in the treatment of adjuvant arthritis in rats make it possible for effective treatment in human arthritis. Clinical trials may be necessary to further explore this potential therapeutic application.

### Summary

These studies utilizing compounds of differing classes, demonstrate that intracellular delivery of cytokine inhibiting drugs to phagocytic cells are extremely effective in cytokine inhibition by increasing intracellular concentrations of the compound. The uptake of microencapsulated drugs by endothelial cells also has great potential in the treatment of many diseases. Microencapsulated drugs of acting to inhibit cytokines by different mechanisms may be additive or synergistic in inhibiting TNF and further improving clinical outcomes. The models of endotoxic shock, peritonitis and arthritis are extreme experimental conditions stimulating cytokines. To our knowledge, there have been no reported results of survival in the peritonitis model of sepsis. The microencapsulated delivery of the antioxidant catalase is also the only data demonstrating the beneficial use of antioxidants in the setting of sepsis. These results document the role that reactive oxygen by-products (H_2_O_2_) contributes to the cytokine activation seen in this condition. Further evaluation of microencapsulated cytokine drugs in conditions which cytokines play an active pathogenetic role in inflammation is warranted in preparation for human clinical trials.
